# Pain Intensity among Community-Dwelling African American Older Adults in an Economically Disadvantaged Area of Los Angeles: Social, Behavioral, and Health Determinants

**DOI:** 10.3390/ijerph16203894

**Published:** 2019-10-14

**Authors:** Meghan C. Evans, Mohsen Bazargan, Sharon Cobb, Shervin Assari

**Affiliations:** 1 Department of Family Medicine, Charles R. Drew University of Medicine and Science, Los Angeles, CA 90095, USA; meg.e.carlsen@gmail.com (M.C.E.); Mohsenbazargan@cdrewu.edu (M.B.); 2 Department of Family Medicine, University of California Los Angeles (UCLA), Los Angeles, CA 90095, USA; 3 School of Nursing, Charles R. Drew University of Medicine and Science, Los Angeles, CA 90095, USA; sharoncobb1@cdrewu.edu

**Keywords:** race, ethnicity, blacks, African Americans, ethnic groups, pain, pain intensity

## Abstract

Background. Although social, behavioral, and health factors influence prevalence and intensity of pain, very few studies have investigated correlates of pain among economically disadvantaged older African American (AA) adults. Objective. This study explored social, behavioral, and health correlates of pain intensity among community-dwelling AA older adults in an economically disadvantaged area of Los Angeles. Methods. A cross-sectional study on 740 AA older adults (age ≥ 55 years) was conducted in South Los Angeles between 2015 and 2018. Exploratory variables were age, gender, educational attainment, financial difficulties, living alone, marital status, smoking, drinking, pain-related chronic medical conditions (CMCs), and depressive symptoms. Dependent variable was pain intensity. Linear regression was used for data analysis. Results. Age, financial difficulties, living alone, smoking, pain-related chronic medical conditions, and depressive symptoms were associated with pain intensity. Individuals with lower age, higher financial difficulties, those who lived alone, those with a higher number of pain-related chronic medical conditions, more depressive symptoms, and nonsmokers reported more pain intensity. Gender, educational attainment, marital status, and drinking were not associated with pain intensity. Conclusion. The results may help with the health promotion of economically disadvantaged AA older adults in urban areas.

## 1. Introduction

Epidemiological studies in the general population have identified several factors associated with pain, such as age, gender, and socioeconomic status (SES) indicators [1,2]. For older adult populations, living alone, depression, smoking, alcohol use, and pain-related chronic medical conditions (CMCs) have been identified as correlates with pain [3,4,5,6,7]. Though research has been able to identify these social, behavioral, and health determinants of pain in general older adult populations, these factors may vary across racial/ethnic groups.

African American (AA) populations are at heightened risk for greater pain intensity, pain sensitivity, pain interference (i.e., the interruption of activities due to pain), and disability [8,9,10,11,12], which are among the main components of pain experiences [11,13,14]. However, AA adults are given less treatment for pain than their White counterparts [15,16]. A review of the literature has attributed the disparity in pain treatment to multiple factors, including patient access and utilization of care, attitudes and beliefs of the patient, and attitudes and beliefs of the provider [17]. Research has documented major mismanagement of pain in older AA adult populations [18]. Current evidence has also revealed false beliefs among White populations and medical students concerning biological differences in AAs, leading to a racial bias in pain perception and treatment [19].

Many social, behavioral, and health factors have been associated with experiences of pain, but such correlates may differ in AAs compared to other racial/ethnic groups. Considering the higher risk of AA populations for pain, the disparity in treatment received, and the increased prevalence of pain conditions that come with aging [1,2], it is vital to understand how various determinants predict pain intensity in this particularly vulnerable population of community-dwelling older AA adults in low-income urban areas. Understanding how these determinants function within this sample will provide evidence on relevant factors to assess when screening for risk and intervention objectives, which is especially vital in an area of limited resources similar to the current study’s sample.

This study aims to expand the existing literature on pain experiences by examining the relationship between pain intensity and a variety of social, behavioral, mental, and physical health factors among community-dwelling older AA adults in a low-income urban area. These factors include gender, age, SES indicators (educational attainment and financial difficulties), living arrangements (living alone and being married), health behaviors (smoking and drinking), depressive symptoms, and pain-related CMCs.

### 1.1. Gender and Age

Previous literature has often found gender differences in pain, such that women are at heightened risk for pain conditions and experience greater pain sensitivity and intensity with lower pain tolerance level [8,20,21]. Even though research has been focused on the heightened pain sensitivity both in women and in AA populations [8], little work has been done on potential gender differences in pain intensity within AA samples.

Age is naturally a factor that has been associated with increases in pain experiences [1,2,22]. Different types of pain have been studied in older adults [6,14,23,24], but less work has been done specifically on the role of age on pain intensity in AA adults [7,25,26,27]. Among the research that has been done, there have interestingly been some findings suggesting that younger age may be associated with greater pain intensity in AA samples [7,26]. Overall, research among both younger and older AA adults indicates pain takes a greater toll on AA quality of life and health than for White adults [25,28]. The complicated associations between age, gender, and pain, especially within AA populations who already have been identified as at risk for increased pain sensitivity and decreased pain treatment, indicates the need to parse out the relationship of these sociodemographic factors within a population of community-dwelling older AA adults.

### 1.2. Socioeconomic Status

Various SES indicators have also been associated with an increased prevalence of pain [13,29]. Research has indicated living in a low socioeconomic area is associated with strong and widespread pain as well as greater disability and distress [30]. Low educational attainment and low income level are also related with greater risk of chronic pain following injury [31], higher pain severity, and greater disability attributed to pain [32,33]. Furthermore, higher education has been associated with more help-seeking behavior in older adults with chronic pain [23]. In terms of financial difficulties, evidence has demonstrated a link between economic hardship and pain severity [34]. Financial strain has been associated with the incidence of pain, while low educational attainment has been associated with the course of pain in terms of intensity and disability [35]. Additionally, a large population-based study by Dorner and colleagues (2011) revealed that given the same severity of pain, those in lower SES groups, stratified by income and education, feel two to three times more disabled by the pain than those in the highest SES group [32].

Recent data highlight the racial/ethnic imbalance in educational attainment and financial difficulties, revealing that 19% of AA young adults have less than a high school education compared to 11% of White adults [36], and 22% of AAs live below the poverty line compared to 8.8% of White adults [37]. Considering these disparities, these SES indicators are likely to be particularly salient to a sample of AA older adults from a low-income area and therefore may be relevant in predicting pain intensity. Although it has been well-established that low SES is associated with health consequences such as increased morbidity [38,39], more research is needed concerning specific SES indicators, such as educational attainment and financial difficulties, and their relationship with pain intensity in older AA samples.

### 1.3. Living Arrangements

Living arrangements among older adults have been studied for their relationship with pain experiences. In a recent study on a large sample of older adults, living alone was associated with the onset of chronic pain for women [4]. However, other evidence has indicated living with a spouse predicted greater pain interference, or pain that interferes with activities, compared to living alone [40]. More help-seeking behavior was also associated with living alone for older adults with chronic pain [23]. Few studies have looked at marital status itself in relation to pain and rather have focused on the influence of certain aspects of the marriage (marital satisfaction, attention, support, etc.) [41]. Some findings indicate that being married in itself is not protective for pain [42], while other research has found significantly lower pain interference for married patients compared to single patients [43]. Overall, relatively little research has been done on whether the factors of living alone or being married can in themselves be significant predictors of pain. Furthermore, even less research has been done specifically for AA samples. Given the role social isolation and a lack of sense of belonging play in the mental distress of older adults who are unmarried or living alone [44,45,46], it will be imperative to understand the relationship these living arrangements have with physical distress for a group of AA older adults who are already at heightened risk for pain experiences.

### 1.4. Health Behaviors

Certain health behaviors, such as smoking and alcohol use, have also been associated with pain [3,6,22,47,48,49]. People may use these types of behaviors in an attempt to cope with pain, and furthermore, health problems tend to co-occur with these behaviors, demonstrating another avenue through which pain intensity and behaviors like smoking and drinking may be linked. Among older adults, smoking and pain are associated, especially in regard to pain intensity [6]. Recent research has identified a mediational pathway such that pain intensity was associated with negative affect, which in turn was associated with the urge to smoke [47]. A recent meta-analysis of the literature on smoking and pain revealed that despite small nicotine-induced analgesic effects, smoking is related to increased chronic pain [50]. Research has also often shown an association between alcohol and pain [3,48,49,51,52]. Furthermore, research has shown that this relationship may be particularly strong for AAs compared to Whites [3,11].

Cumulatively, the research described above suggests a potential cyclical relationship such that pain promotes the use of cigarettes and alcohol in an attempt to suppress pain. However, the continued use and excessive dependence on cigarettes and alcohol could lead to pain-related CMCs and increased pain experiences. This relationship seems especially relevant among AA populations who have an increased risk for pain, as well as increased exposure to other stressors, such as financial difficulties, which are also associated with smoking and alcohol consumption [53,54]. However, more research is needed to understand the relationship between these health behaviors and pain intensity, as most research has been on predominantly White samples [3,6,47,51].

### 1.5. Depressive Symptoms

A recent meta-analysis revealed that pain is significantly related to psychological issues, including depression [55]. In terms of racial/ethnic differences, research also indicates pain intensity is associated with greater depressive symptoms for AAs compared to Whites [25]. Additionally, a study on older AA women revealed that more depressive symptoms significantly predicted greater pain intensity in that sample [7]. Depressed older adults compared to nondepressed older adults more often report chronic pain and as well as the pain being more severe and disabling [14]. Furthermore, a recent study examining pain in a sample similar to the current study—community-dwelling older AA adults in south Los Angeles—found severe pain to be associated with higher levels of depression [56].

Given the evidence for the relationship between depressive symptoms and pain intensity, especially in older adults and in AA adults [7,14,25,55,56], it is necessary to include depressive symptoms in our model of potential predictors for pain intensity within this sample of older AA adults.

### 1.6. Pain-Related CMCs

As can be expected, pain-related CMCs, such as rheumatoid arthritis and fibromyalgia, are naturally related to pain [52,57]. A recent study underlined the importance and prevalence of the comorbidity of multiple pain-related CMCs, such as musculoskeletal conditions and microvascular disease, in predicting pain in older adults [5]. There is considerable evidence for racial/ethnic disparities in pain-related CMCs showing that AAs are at increased risk for multiple CMCs [58,59,60].

Additionally, research in a sample of older AA adults in Los Angeles, similar to the current study, found evidence for severe mismanagement of pain, especially for those with multiple pain-related CMCs [18]. Given the heightened prevalence of CMCs in AA populations, especially among those who are aging, it will be particularly important to determine the relationship between pain-related CMCs and pain intensity in our community-dwelling sample from an underserved area that may not have access to proper diagnosis or treatment.

### 1.7. Current Study

This study examined the association of gender, age, SES indicators (educational attainment and financial difficulties), living alone, marital status, smoking, drinking, depressive symptoms, and pain-related CMCs with pain intensity in community-dwelling AA older adults in an economically disadvantaged area of Los Angeles. Based on the previous literature, we hypothesize that being a woman, older age, lower educational attainment, more financial difficulties, living alone, being unmarried, drinking, smoking, greater depressive symptoms, and more pain-related CMCs would be associated with higher pain intensity. Considering that most of the previous work on these determinants and pain intensity was conducted among the general population, this study aimed to uncover the relationship between these social, behavioral, and health factors among an older AA population in an economically disadvantaged setting.

## 2. Methods

### 2.1. Design and Setting

Between 2015 and 2018, a cross-sectional survey was performed with a sample of community-dwelling older AA adults from an economically disadvantaged area in South Los Angeles.

### 2.2. Institutional Review Board (IRB)

The Charles R. Drew University of Medicine and Science (CDU) Institutional Review Board (IRB) approved the study protocol. All participants signed a written informed consent prior to their enrollment and received a financial incentive.

### 2.3. Process and Data Collection

Structured face-to-face interviews were used which included several pages of checklist-style questionnaires composed by the investigator based on previous research as well as the standard validated measures cited below and a medication evaluation. The interviews took place over several hours and were conducted by physicians who were members of the research team in private rooms in churches or apartments near the areas where participants were recruited. Through structured interviews and the self-reported checklist-style questionnaires, data were collected concerning demographic factors (gender and age), SES (educational attainment and financial difficulties), living alone, marital status, health behaviors (smoking and drinking), depressive symptoms, pain-related CMCs, and pain intensity. The primary study included a comprehensive evaluation of the medication used by participants which was not included in the current study.

### 2.4. Participants

Community-dwelling older AA adults were recruited through convenience sampling from 16 predominantly AA churches, 11 senior residential units, and low-income public housing projects in South Los Angeles. Recruitment was facilitated through church leaders and apartment managers who referred eligible adults in their community to participate. Participants were told the study was a comprehensive health evaluation and would include collecting demographic, social, behavioral, and health information. Adults were eligible to participate if they were AA, 55 years or older, could complete an interview in English, and resided in Service Planning Area 6 (SPA 6). SPA 6 was prioritized as the setting for this study because it is one of the most economically disadvantaged urban areas in the Los Angeles county, with the highest percentage of household incomes less than 100% of the Federal Poverty Level (33.6%) and the highest unemployment rate (13.6%) [61,62]. Additionally, SPA 6 has the highest percentage of AAs (27.4%) of the eight SPAs in Los Angeles [61].

Having a chronic pain diagnosis or medical condition was not part of the eligibility criteria for this sample as the goal of the study was to measure pain intensity for a community-dwelling sample rather than a clinical sample. Institutionalized adults and those enrolled in other clinical trials were excluded. Through the process of recruitment and consent, interviewers assessed that there was no profound cognitive deficit that would prohibit the participant from completing the task. This resulted in a sample of 740 AA adults aged 55 or older.

### 2.5. Measures

The current study collected data on demographic factors (gender and age), SES (educational attainment and financial difficulties), living alone, marital status, health behaviors (smoking and drinking), depressive symptoms, pain-related CMCs, and pain intensity.

#### 2.5.1. Dependent Variable

*Pain Intensity.* Pain intensity was measured using four subscales of the McGill Pain Questionnaire– Short Form 2 (MPQ-SF-2) [63,64,65,66]. This consisted of 22 items which asked participants to rate the extent of their past-week experience of various types of pain on an 11-point numeric scale (0 none to 10 worst possible). The subscales of the MPQ-SF-2 include: (a) continuity (throbbing, cramping, gnawing, aching, heavy, and tender pain), (b) intermittence (shooting, stabbing, sharp, splitting, electric-shock, and piercing pain), (c) neuropathic nature (hot-burning, cold-freezing, itching, tingling or “pins and needles”, light touch, and numbness pain), and (d) affective domain (tiring-exhausting, sickening, fearful, and punishing-cruel pain). Based on the average responses to all questions, a total pain score was calculated [63,64,65,66], and more intense pain was indicated by a higher score (α = 0.945).

#### 2.5.2. Independent Variable

*Demographic Factors.* Gender was a dichotomous variable (1 female, 0 male), while age was operationalized as a continuous variable. This information was collected through self-report checklist-style questionnaires.

*Socioeconomic Status (SES).* Educational attainment was treated as a continuous variable (years of schooling), with higher educational attainment reflected by higher scores. Multiple systematic reviews on the relationship between educational attainment and various health factors have cited years of education as a common measurement tool [67,68,69]. We measured financial difficulties using three items (α = 0.923) that asked how often participants lacked enough money for necessities like food, rent/mortgage, clothes, and utility bills. Responses for each item were given on a 5-point Likert scale (1 never to 5 always), and a sum score was built ranging between 3 and 15, with more financial difficulties (lower SES) indicated by higher scores. The items used were consistent with Pearlin’s list of chronic financial difficulties experienced by individuals of low SES [70]. This information was collected through self-report checklist-style questionnaires.

*Living Arrangement and Marital Status.* Living arrangements and marital status were treated as dichotomous variables (1 living alone, 0 living with someone else; 1 married, 0 not-married). This information was collected through self-report checklist-style questionnaires. Previous research on pain and marital status has also utilized this method of dichotomous measurement [71,72], Additionally, systematic reviews indicate living alone have often been assessed in a similar way in research concerning older adults [73,74].

*Health Behaviors (Smoking and Drinking).* Participants reported their current smoking and drinking status by answering questions such as “How would you describe your cigarette smoking habits?” and “Do you drink alcohol?” Responses to the first question included never smoked, previously smoked, and current smoker. Responses to the second question were yes or no. Both variables were treated as dichotomous variables (1 ever smoker, 0 never smoker; 1 drinker, 0 nondrinker). While there is evidence for the validity of self-report measures of smoking and drinking, some self-report bias may be expected [75,76].

*Depressive Symptoms.* Depressive symptoms were measured using the 15-item short Geriatric Depression Scale (GDS) [77,78]. Responses were on a “yes” or “no” scale. Summary scores were calculated, ranging between 0 to 15, with more depressive symptoms indicated by higher scores. The GDS-Short form has been used extensively to measure depression among older adults in community and clinical settings and has shown excellent reliability and validity [77,78].

*Pain-Related CMCs.* Participants reported whether a physician has ever told them that they have any of the following CMCs: cancer, gastrointestinal disease, osteoarthritis, and rheumatoid arthritis. Even though self-reports provide valid information regarding CMCs, some level of measurement bias is expected [79].

### 2.6. Statistical Analysis

We used SPSS 22.0 (SPSS Inc., Chicago, IL, USA) to conduct the data analysis. Frequency (%) and mean (SD) were reported describing the sample. We used Pearson’s correlation to calculate the bivariate correlations in the overall sample. Linear regression models were applied with pain intensity as the outcome, and the independent variables, entered simultaneously, were gender, age, educational attainment, financial difficulties, living alone, marital status, smoking, drinking, depressive symptoms, and pain-related CMCs. The list of predictors was determined based on the literature review. Correlation coefficients (b), standard error (SE), 95% confidence intervals (95% CI), and *p* values were reported.

## 3. Results

### 3.1. Descriptive Statistics

This study included 740 low-income AA older adults who were 55 years or older. Most participants (*n* = 474) were women. Table 1 describes the sample descriptive statistics.

### 3.2. Bivariate Correlations

Table 2 shows the summary of bivariate (zero order) correlations between the study variables. As this table shows, age, financial difficulties, marital status, living alone, ever smoking status, drinking, depressive symptoms, and number of pain-related CMCs were all correlated with pain intensity.

### 3.3. Linear Regression

As Table 3 shows, age, financial difficulties, living alone, smoking, pain-related CMCs, and depressive symptoms were associated with pain intensity. Individuals who were younger, those with higher financial difficulties, individuals who lived alone, nonsmokers, individuals with a higher number of pain-related CMCs, and individuals with more depressive symptoms reported more pain intensity. Gender, educational attainment, marital status, and drinking were not associated with pain intensity.

## 4. Discussion

Age, living arrangement, financial difficulties, smoking status, depressive symptoms, and pain-related CMCs were significantly correlated with pain intensity among a sample of older AA adults living in an economically disadvantaged setting. Individuals who were on the younger side of the age range, higher financial difficulties, individuals who lived alone, nonsmokers, individuals with higher number of pain-related CMCs, and individuals with more depressive symptoms reported greater pain intensity. However, gender, educational attainment, marital status, and drinking were not associated with pain intensity.

### 4.1. Age, Gender, and Pain Intensity

While much of the research in the broader population would suggest that older age would predict higher pain intensity [1,2,22], our findings demonstrate that within this AA sample, younger age was associated with greater pain intensity. Even though research comparing across races has found that older AA adults are at higher risk for pain than older White adults [25,27], research comparing the effects of age within AA samples have found that younger age was associated with more pain intensity [7,26], which aligns with our findings. Baker and colleagues attributed the finding of higher pain intensity in younger AA adults to the possibility of the crossover effect, which suggests minority members who reach old age have built up physical and psychological resilience as well as effective coping methods, providing them with some protection their younger or majority race/ethnicity counterparts may not have [7,80]. More research is needed to parse out the nuances and mechanisms of the relationship between age and pain in the AA population.

Similarly, gender differences have often been found across pain metrics indicating women are more likely to have higher pain intensity [8,20,21], yet our results show gender did not significantly predict pain intensity in our sample. This may align with more recent research which found that when women and men experience the same level of pain severity, women have higher levels of activity and pain acceptance than men [81]. Furthermore, in a study of older adults, women were more likely to seek help for chronic pain [23]. Therefore, it is possible that women may handle and seek out treatment for pain differently than men which may have been overlooked in previous studies. Furthermore, it is possible the women in this study handle pain particularly well given the majority of participants were women and the average pain score was relatively low. Even though there are multiple studies demonstrating the heightened risk of pain predicted by gender and race independently [1,2,10,25], relatively little work has examined the role of gender within a racial/ethnic minority group. Perhaps the older AA women in this study have a particular resilience to pain following a similar theory as the crossover effect cited above [7,80], which suggests coping methods as well as physical and psychological resilience accumulate over time among minority members. According to the interaction theory of race/ethnicity and gender, older AA women hold multiple subordinate identities in society, and therefore, their experiences of disparities are compounded [82,83]. Perhaps the AA women who have reached older age have accumulated specific skills or resiliencies in order to deal with physical and psychological pain, and therefore, the typical gender differences in pain intensity do not appear in our findings. Future research should seek to replicate these findings in other samples of older AA adults in order to understand if gender differences in pain vary by race/ethnicity or age. The current study adds to the literature by demonstrating that within a sample of low-income older AA adults, gender does not significantly correlate with pain intensity as one may expect based on research conducted in the broader population.

### 4.2. SES and Pain Intensity

Of the two SES indicators measured in this study, financial difficulties were associated with pain intensity, while educational attainment was not. Despite previous research which suggests that low SES, specifically lower educational attainment, is associated with more pain [31,32,33], the current findings align with other recent research which indicates educational attainment does not provide the same protective benefits for AA populations compared to Whites [84,85]. This research, along with our findings, provides support for the minorities’ diminished returns (MDR) theory which suggests minority populations such as AAs receive smaller health gains as their SES increases compared to Whites [86]. The current findings further our understanding of the MDR theory by showing educational attainment was not associated with pain intensity within this population of older AA adults in a low socioeconomic area.

On the other hand, financial difficulties were associated with higher pain intensity for this sample. This aligns with past research findings which have linked financial strain to greater risk for chronic pain following injury [31], pain incidence [35], and pain severity [32]. Given the disparity between AAs living below the poverty line compared to Whites [37], it is understandable that financial difficulties may be an especially relevant predictor of increased pain intensity in our sample of older AA adults from an economically disadvantaged area. This finding sheds further light on an important risk factor for well-being in AA populations like this.

### 4.3. Living Arrangements and Pain Intensity

Our findings revealed that living alone but not marital status predicted pain intensity. While some research has found that for older adults, living alone was associated with pain onset [4], other research has indicated marital status and certain factors about the marriage (i.e., spouse attention, marital satisfaction) could be more predictive of pain [40,41]. Given these complicated findings, the current research adds to the literature on living arrangements and pain specifically for AA adults—a population where little research has been done on this subject. Our findings demonstrate that those who were living alone were at risk for higher pain intensity.

Given previous findings that social isolation is related to health, such that it is associated with higher stress intensity and less efficacious physiological repair and wound-healing [44], it is understandable how living alone could influence pain intensity. Furthermore, there is an increasing amount of evidence for the role of loneliness in pain, with recent longitudinal work indicating social isolation can predict increases in future pain interference [87]. Additional research has found pain intensity and pain frequency are associated with greater loneliness and worse social functioning [88]. Our findings indicating the relationship between living alone and pain intensity may best be explained within the context of these potential mediating factors. Additionally, other social factors not measured in the current study which may protect against pain intensity could be harder to access for older AA adults living alone. Family support, for example, is related to less pain intensity, higher activity levels, and less reliance on pain medication [89]. Furthermore, our results showing a lack of association between marital status align with other research which has shown qualities of the marriage itself rather than marital status alone are better predictors of pain [41,42]. Future research should continue to explore the aspects of living alone that could mediate the relationship found in our results as well as the aspects of marriage that may act as risk or protective factors for pain in low-income older AA adults.

### 4.4. Health Behaviors and Pain Intensity

Past research indicates a relationship between both smoking and drinking and pain [3,6,22,48,49,90]. Our findings did indicate an association between pain intensity and nonsmoking but no association between pain intensity and drinking in our sample of older AA adults. We might expect a cyclical relationship such that people use these types of health behaviors in an attempt to dampen pain intensity, but increased frequency of these health behaviors, in turn, leads to other chronic issues and potentially increased pain [48,52]. Past research has demonstrated this association in AA populations [90,91], even showing a stronger relationship between pain and substance use for AAs compared to Whites [3,11].

While those with more pain may cite smoking as a coping strategy, research has found smoking cigarettes is associated with greater pain intensity and pain interference [92]. Other research indicates that smokers may report having a need to smoke but also show significantly higher levels of emotional distress and rely on medication more than nonsmokers [93]. A recent meta-analysis attempted to clarify this complex literature on pain and smoking, and found that, while smoking can provide a small acute nicotine-induced analgesia which may make smoking rewarding, current smoking is associated with more severe pain [50]. Our findings that nonsmoking was related to increased pain intensity, therefore, do not align with this past research. There is, however, some research specifically in AA samples, indicating smoking is linked to less pain intensity and smoking abstinence is associated with increased acute pain [90,91], which would align with our current findings. Future research should explore whether this relationship between nonsmoking and pain intensity is unique to AA populations. Furthermore, it is possible that those in who were classified as “ever smokers” are made up mostly of previous rather than current smokers, considering current smokers would be less likely to reach older age and therefore be a part of our sample, and this may at least partially explain why we did not find the typical association between smoking and pain.

Previous research has indicated a relationship between pain interference and alcohol abuse or alcohol-related problems that is stronger for AAs compared to Whites [3,11]. However, moderate alcohol consumption is related to pain reductions, suggesting a potential curvilinear relationship between moderate alcohol consumption and pain improvement but deleterious pain outcomes related to excessive drinking [48,52]. However, our findings of no association between alcohol use and pain intensity do not align with past research. It is possible that our measurement of alcohol use may have prevented us from the detection of this relationship. The current study did not measure alcohol use disorder (AUD) or alcohol dependence but only current drinking status. It is possible that if the study had more data on the level of these health behaviors, a higher severity of drinking might have revealed the expected relationship found in other literature.

### 4.5. Depressive Symptoms and Pain Intensity

As expected, based on previous literature, our findings indicate depressive symptoms are positively associated with pain intensity. The Black–White paradox, however, proposes that even though AA populations are exposed to greater distress and health issues, they display lower rates of mental disorders such as depression, suggesting that the pain–depression link may be weaker than expected in AA older adults [94]. The current findings run contrary to the Black–White paradox, however, and expand the understanding of mental well-being in AAs by taking into account depressive symptoms regardless of disorder diagnosis. This is imperative considering that past research has indicated disparities in diagnosis of depression in AAs [95] and higher chronicity and severity of depressive symptoms for AAs compared to Whites [96].

Our findings align and add to past research which has indicated pain intensity is associated with greater depressive symptoms in AAs and older adult samples [7,14,25,56]. This research helps to clarify the Black–White paradox theory surrounding the mental resilience of AA populations when exposed to distress such as pain intensity. Furthermore, it highlights depressive symptoms as a significant predictor of pain intensity in a sample of older AA adults, specifically from a low-income area who have fewer access to not only physical but also mental health resources.

### 4.6. Pain-Related CMCs and Pain Intensity

As predicted, pain-related CMCs were strongly correlated with pain intensity in our sample. Research on pain intensity has been studied in relation to a variety of medical conditions, both those that are typically pain conditions and those that are nonpain conditions [5,52,57]. Including pain-related CMCs as a predictor of pain is especially relevant in our sample, given that AAs are at heightened risk for a variety of CMCs that may or may not be associated with pain [58,59,60].

Demonstrating the relationship between pain-related CMCs and pain intensity in this sample of older AA adults in a low-income area is especially relevant given that aging AAs are at heightened risk for CMCs, and the area itself may lack resources for treatment for these conditions. Furthermore, research has shown low-income older AA adults experience severe mismanagement of pain, especially when they have multiple pain-related CMCs [18]. This research further highlights the necessity to focus on pain-related CMCs in these underserved populations in order to address the risk for pain intensity.

### 4.7. Bivariate Correlations

In addition to the results above yielded from our linear regression analysis, we also gained important information from our bivariate correlation analysis. While our linear regression adjusted for covariates, the bivariate correlation analysis revealed the unadjusted relationship between variables. Through this analysis, depressive symptoms and CMCs were identified as two variables which were most strongly associated with pain intensity. As stated above, this naturally aligns with our hypotheses as well as previous research on these variables. Depressive symptoms have been linked to pain [55], and this relationship appears especially strong in AA and older adult samples [7,14,25,56]. Furthermore, CMCs are understandably strongly related to pain [5,57], and AAs specifically are at heightened risk for a variety of CMCs [58,59,60]. These results, along with the linear regression results, add to the literature by showing the strength of this relationship in a sample at the intersection of multiple previously studied demographics (i.e., older AA adults in a low-income setting). Furthermore, it demonstrates the necessity to consider these physical and mental health conditions when screening for and focusing on interventions towards risk for pain intensity.

### 4.8. Limitations

This study is not without limitations. Causal associations cannot be inferred due the cross-sectional design of the study. Furthermore, this study did not have data on area level factors, household income, or history of depression, nor did it use clinical records to verify CMCs or mental health diagnosis. Thus, self-report (measurement) bias may exist. Furthermore, our measures of drinking and smoking did not take into account the frequency or quantity of consumption which could provide additional understanding about the relationship between these factors and pain intensity. Future research should use administrative data, medical chart review, and additional health behavior measures to attempt to replicate and expand on these findings. Our measures of marital status did not take into account a variety of other possible relationship statuses, such as long-term partnership, divorce, or widowhood. Our dichotomous measure of living alone also did not measure social support that comes outside of cohabitation. These various other aspects of relationship status and living arrangements could provide more insight into aspects of pain intensity for this sample and should be implemented in future research. The pain intensity measure was designed to assess the intensity of various types of pain within the last week and therefore did not assess the frequency or persistency of this pain. Considering pain intensity is just one component within the concept of pain experiences, future research should explore the relationship of these variables with other aspects of pain, such as frequency, site of pain, and pain interference.

Additionally, generalizability of these results cannot be guaranteed due to the nonrandom sampling. This sample was taken from one location and was made up of mostly women, which reduces the generalizability of these findings as well. Notwithstanding these limitations, this study provides important evidence furthering our understanding of determinants of pain intensity among older AA adults within a low-income urban setting.

## 5. Conclusions

Age, financial difficulties, pain-related CMCs, living alone, smoking status, and depressive symptoms are all significantly associated with pain intensity for older AA adults in a low-income area of Los Angeles. Future research should also focus on discovering the most effective ways to reduce the burden of pain in older AA samples. Given the limited resources in low-income urban areas, these determinants should be prioritized as factors to be screened for in order to identify older AA adults at risk for heightened pain intensity. Clinical interventions may also address these factors as a means to improve overall well-being of AA older adults through reducing risk for pain intensity.

## Figures and Tables

**Table 1:** Descriptive statistics in the pooled sample (*n* = 740).

Characteristics	*n*	%
Gender		
Women	474	64.1
Men	266	35.9
Living Arrangements		
Living with others	294	39.7
Live alone	446	60.3
Ever Smoker		
No	347	47.0
Yes	391	53.0
Drinking Alcohol		
No	481	65.1
Yes	258	34.9
Marital Status		
Other	640	86.5
Married	100	13.5
	Mean	SD
Age	71.7	8.4
Educational Attainment	12.7	2.2
Financial Difficulties	8.9	5.5
Number of pain-related CMCs	1.6	1.2
Depressive symptoms	2.5	2.8
Pain Intensity	2.0	2.3

SD: Standard deviation; CMCs: Chronic medical conditions.

**Table 2:** Bivariate correlations.

Characteristics	1	2	3	4	5	6	7	8	9	10	11
1 Age	1	−0.08*	−0.18**	−0.31**	−0.00	0.06	−0.27**	−0.21**	−0.08*	−0.25**	−0.23**
2 Gender		1	−0.11**	0.07	0.12**	−0.09*	0.17**	0.04	−0.17**	0.02	−0.07
3 Educational Attainment			1	−0.08*	0.06	−0.04	−0.06	0.09*	−0.08*	−0.07	−0.02
4 Financial difficulties				1	−0.07*	0.12**	0.31**	0.16**	0.27**	0.43**	0.36**
5 Married					1	−0.41**	−0.06	−0.08*	−0.05	−0.07	−0.08*
6 Living alone						1	0.12**	0.08*	0.12**	0.12**	0.16**
7 Smoking							1	0.24**	0.17**	0.23**	0.14**
8 Drinking								1	0.07	0.08*	0.12**
9 Pain-related CMCs									1	0.36**	0.54**
10 Depressive symptoms										1	0.46**
11 Pain intensity											1

SE: Standard error; 95% CI = 95% Confidence interval; CMCs: Chronic medical conditions; * *p* < 0.05, ** *p* < 0.01.

**Table 3:** Multivariable model of determinants of pain intensity in a sample of economically disadvantaged AA older adults in South Los Angeles.

Characteristics	B	SE	95% CI	*p*
Age	−0.03	0.01	(−0.05, −0.01)	0.001
Gender	−0.01	0.14	(−0.29, 0.28)	0.967
Educational attainment	0.02	0.03	(−0.04, 0.08)	0.594
Financial Difficulty	0.04	0.01	(0.02, 0.07)	0.001
Married	−0.07	0.21	(−0.48, 0.35)	0.749
Living alone	0.36	0.15	(0.07, 0.65)	0.015
Ever Smoker	−0.31	0.15	(−0.60, −0.03)	0.032
Drinking alcohol	0.20	0.15	(−0.09, 0.49)	0.175
Pain-related CMCs (n)	0.80	0.06	(0.68, 0.92)	0.000
Depressive Symptoms	0.19	0.03	(0.13, 0.24)	0.000
Constant	1.70	0.87	(−0.01, 3.42)	0.052

SE: Standard error; 95% CI = 95% Confidence interval; CMCs: Chronic medical conditions.
